# Primary, Non-Refluxive Megaureters: Ureteral Peristalsis Detected on Initial Ultrasound Is Not a Valid Predictor for Spontaneous Resolution

**DOI:** 10.3390/children13040524

**Published:** 2026-04-09

**Authors:** Christa Gernhold, Franziska Rameseder, Lukas Steinkellner, Bernhard Haid

**Affiliations:** 1 Department of Pediatric Urology, Hospital of the Sisters of Charity, 4020 Linz, Austria; franziska.rameseder@ordensklinikum.at (F.R.); lukas.steinkellner@ordensklinikum.at (L.S.); bernhard.haid@ordensklinikum.at (B.H.); 2 Department of Urology, University Hospital Salzburg, Paracelsus Medical University, 5020 Salzburg, Austria

**Keywords:** primary megaureter, peristalsis, ultrasound

## Abstract

**Highlights:**

**What are the main findings?**

• Sonographically visible ureteral peristalsis at initial diagnosis does not predict spontaneous resolution, pyelonephritis, or the need for surgery in primary non-refluxing megaureters.
• Ureteral peristalsis is a highly variable and inconsistent finding during ultrasound follow-up.

**What are the implications of the main findings?**

• The initial detection of ureteral peristalsis should not influence clinical decision-making in infants with primary non-refluxing megaureters.
• There remains a need for reliable, non-invasive predictors to guide follow-up intensity and the timing of intervention.

**Abstract:**

**Background/Objectives:** Primary non-refluxing megaureters (PM) are common congenital anomalies of the urinary tract. While spontaneous resolution is frequent, reliable non-invasive predictors of outcome are scarce. Ureteral peristalsis is frequently regarded as a sign of functional maturation and favorable prognosis, although supporting clinical evidence is limited. This study aimed to evaluate whether sonographically visible ureteral peristalsis at initial diagnosis predicts spontaneous resolution, pyelonephritis, or the need for surgery in infants with PM. **Methods:** In this retrospective single-center study, infants diagnosed with primary non-refluxing megaureters before one year of age between 2012 and 2018 were analyzed. Patients with refluxing, secondary, syndromic, or ectopic megaureters were excluded. Sonographic detection of distal ureteral peristalsis at initial examination was recorded. Clinical outcomes included spontaneous resolution, episodes of pyelonephritis (including breakthrough infections under antibiotic prophylaxis), and surgical intervention. Univariate and multivariate logistic regression analyses were performed. **Results:** Sixty-three infants were included, with a median follow-up of 34 months. Peristalsis was detected in 52.3% at initial ultrasound. Complete spontaneous resolution occurred in 66% of patients, while 20.9% required surgical reimplantation. The presence of peristalsis at diagnosis was not associated with spontaneous resolution, time to resolution, occurrence of pyelonephritis, breakthrough infections, or surgical intervention. Multivariate analysis confirmed that initial peristalsis was not an independent predictor of outcome. **Conclusions:** Sonographically visible ureteral peristalsis is a transient and inconsistent finding in infants with primary non-refluxing megaureters and does not predict clinical outcome. Peristalsis observed on initial ultrasound should not be used as a decision-making parameter in the management of PM during the first year of life.

## 1. Introduction

Primary non-refluxing megaureters (PM) are common among congenital malformations of the urinary system [1]. PM are defined as >7mm in prevesical diameter. Regarding their grade of dilatation and their morphology, three types are differentiated according to Pfister and Hendren, although with little prognostic impact [2,3,4]. Only recently has the threshold of 7 mm in retrovesical diameter been shown to be significantly associated with a higher risk of febrile urinary tract infections (fUTIs) [5]. Setting aside the increased risk of UTIs, mostly during the first year of life, prognosis regarding spontaneous resolution is favorable, with a spontaneous resolution rate of up to 80% [1]. Indications for surgery include recurrent fUTIs, progressive or persistent dilatation on ultrasound, differential renal function (DRF) <40% or a significant decrease in DRF of ≥5%, nephro- or ureterolithiasis, and (rarely) flank or abdominal pain.

Clinical guidelines emphasize the degree of dilation and renal function as determinants of risk and management strategy, not peristalsis. Therefore, peristalsis is not routinely used as a prognostic marker in clinical practice, and a standardized assessment of peristalsis for outcome prediction is lacking in the literature.

Early on, the notion about an aperistaltic distal segment of a megaureter as a defining pathophysiological detail was presented, based on radiological studies [6]. The pathophysiology of occurrence, and especially the mechanisms involved in the postnatal resolution of megaureters, are widely unclear. Pirker et al. showed that the intramural parts of the ureter mature only in the third trimester of gestation in terms of their muscular layers, and continue to evolve during the first two years of life [7].

Also, peristalsis of the ureter might play a role in its functional maturation: while there is little clear evidence, a lack of physiological peristalsis in a distal segment of the potentially not-yet-developed ureter is widely accepted as a potential reason. In a very recent mathematical modeling study, peristalsis was brought into the center of an etiological theory about megaureters demonstrating the effects of a malfunction in longitudinal muscle fiber action on ureteral dilation. The authors conclude that the presence of peristalsis prevents ureteral dilatation [8].

Megaureters with present, functional peristalsis might therefore confer more efficient urine transport compared to ureters without peristalsis. It has been shown in the literature that dilatation actively affects the electrophysiology of the ureteral wall and its subsequent peristalsis [9,10,11]. To the best of our knowledge, there is, at present, no research available that presents an evaluation of the physiological appearance of ureteral peristalsis on ultrasound in children with ureteral dilation

This study aims to evaluate the predictive power of visible peristalsis on initial ultrasound as a non-invasive, readily available test regarding the odds of spontaneous resolution, pyelonephritis and breakthrough pyelonephritis during antibiotic prophylaxis.

## 2. Materials and Methods

All children treated between 2012 and 2018 at the Department of Pediatric Urology with a ureteral diameter greater than 7 mm, as measured by ultrasound, were considered for inclusion in this retrospective, single-institution review study. After obtaining the study protocol obtained clearance from the university ethical review board (JKU-EK 1300/2022), several exclusion criteria were applied to reduce heterogeneity and focus on primary non-refluxing megaureters. Children were excluded if they were older than 1 year at the first appointment or if there was diagnostic misclassification with a ureteral diameter below 7 mm. Further exclusions were due to reflux-associated megaureters, ureteroceles, ectopic ureters, and secondary causes of ureteral dilation, including posterior urethral valves, tumors, or urinary tract stones and those associated with ureteroceles. After applying these criteria, 63 patients remained for definitive analysis. In addition to the demographical data, characteristics of the megaureters, including their retrovesically measured width in a transverse section in consecutive exams, as well as the characteristics of the upper tract, including data from MAG3 examinations, were recorded. Furthermore, data on the occurrence of UTIs under or without prophylaxis was noted. Finally, the further course over all recorded follow-up exams and eventual surgical interventions were assessed.

A sonography of the bladder and kidneys was performed by pediatric urologists with a high level of experience (>500 exams/year over at least 10 years) in sonography. Patients underwent initial follow-up ultrasound examinations at 3-month intervals. Thereafter, the frequency of imaging was adjusted according to the individual clinical course.

The presence of peristalsis was defined as clearly visible distal ureteral contractions during the examination, lasting 20–30 s, with at least partial coaptation of the ureteral walls, regardless of initial ureteral diameter as recorded in the first examination note. Although variability is inherent to ultrasound, observations of clear peristaltic activity within 30 s were sufficient. Conversely, if no movement is detected in a dilated ureter during the assessment, it can be reasonably assumed that peristalsis is absent. While a fully standardized ultrasound protocol—including defined hydration—could improve reproducibility, such a protocol is not practical in routine clinical settings.

While the variability inherent to ultrasound examinations is a well-recognized phenomenon, the parameter examined in this study was intentionally designed to reflect real-world clinical assessments [12].

Voiding cystourethrography and MAG3 scans were carried out in accordance with our departmental standards [13,14]. Diuretic renography was performed in accordance with international standards. Prior to the examination, all patients received a standardized intravenous fluid regimen (50 mL/h over 3 h, ELO Paed balanced with 1% glucose^®^, Fresenius Kabi, Bad Homburg, Germany) and maintained a normal diet.

After at least one hour of standardized hydration, 99 mTechnetium-labeled MAG3 was administered intravenously. Furosemide (1 mg/kg, maximum 20 mg) was injected intravenously according to the F + 20 protocol. Standard imaging techniques were employed: flow images were acquired at 1 s per frame for the first 3 min to evaluate renal perfusion and initial drainage, followed by 20 s per frame images over 27 min to assess diuretic-induced clearance.

The basic renogram consisted of a dynamic phase with a minimum duration of 30 min for F + 20a, a post-micturition image obtained after a mean of 45 min, and, in cases with <50% residual activity on the post-micturition image, a late image at 2 h post-injection. This imaging sequence allowed for a comprehensive assessment of renal function, ureteral drainage, and potential obstruction. Vesicoureteric reflux into a megaureter was defined as any retraceable contrast agent in anterioposterior or lateral views.

UTI was defined as a positive urine culture from catheterized urine >10^5^ CFU (colony forming units) containing uropathogenic bacteria in symptomatic (febrile) children.

Follow-up exams, including a sonography, were routinely scheduled at three-monthly intervals during the first year of life, and then every 6 months thereafter.

Surgery was indicated after recurrent episodes of pyelonephritis with or without antibacterial prophylaxis or loss of renal function in consecutive MAG3 scans with a decline in DRF ≥5% on the ipsilateral side.

Data was assessed from the hospital information system (SAP, Walldorf, Germany), pseudonymized and collected in a Microsoft Excel database. Relevant parameters were analyzed using descriptive statistics, univariate testing (Fisher’s Exact Test) using Prism 10 (Graphpad Software, Boston, MA, USA) and a stepwise multivariate logistic regression analysis performed with the R statistical package (R Core Team 2023. _R: A Language and Environment for Statistical Computing_. R Foundation for Statistical Computing, Vienna, Austria) (Prism 10 for Mac OS X, Version 10.6.1 (799), 8 September 2025; R version 4.5.1 (2025-06-13 ucrt, Great Square Root) https://www.R-project.org/).

## 3. Results

A total of 308 infants admitted to our department for further evaluation of suspected megaureter were initially screened. Inclusion required a retrovesical ureteral diameter greater than 7 mm, as measured by ultrasound.

To reduce heterogeneity and focus on primary non-refluxing megaureters, several exclusion criteria were applied. Patients who presented after the age of one year were excluded (*n* = 133), as were cases of diagnostic misclassification with a ureteral diameter below the 7 mm threshold (*n* = 5). Further exclusions included syndromic or unclear underlying conditions such as Prune Belly syndrome (*n* = 2), reflux-associated megaureters (*n* = 38), ureteroceles (*n* = 26), ectopic ureters (*n* = 31), and secondary causes of ureteral dilation such as posterior urethral valves, tumors, or urinary tract stones (*n* = 10).

After applying these criteria, a final cohort of 63 infants with primary non-refluxing megaureters diagnosed before the age of one year and with available follow-up data were included for analysis.

The group of 63 patients presented at a median age of 1.57 months; the median duration of follow-up was 34 months. In 39% (25/63) of all included patients, a dilated pelvicalyceal system or a dilated ureter was diagnosed pre-partum, while 9% (6/63) were diagnosed after a first UTI. Duplex kidneys without VUR into the respective lower-pole ureter were present in 3% (2/63). In 18% (13/63), contralateral pathologies of the urinary tract (hydronephroses, duplex systems) were present. In all patients, antibiotic prophylaxis was prescribed, but 12% (8/63) of parents refused to give the medication. At the initial diagnosis, none of the boys were circumcised. Patient characteristics are summarized in Table 1.

Of the 63 included children, 46 underwent a total of 107 MAG3 scintigraphies (1–8; median 2; mean 2.34) during this period. The mean ipsilateral renal function was 47.3%. A persistent obstructive outflow curve during follow-up was observed in 10.8% (5/46) of patients, with a preserved split function on last MAG 3 (DRF 45%).

**Figure 1:**
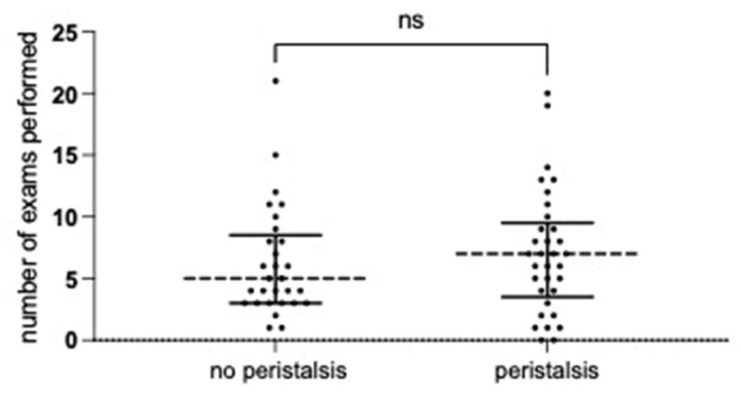
Number of follow-up exams in patients with or without detection of initial peristaltic. The dashed horizontal lines indicate the median values, the solid lines indicate the interquartile range for each group.

The cohort underwent a substantial number of diagnostic and follow-up investigations. A total of 445 ultrasound examinations were performed, with up to 21 follow-up ultrasounds recorded per patient (median: 7 in the group with detectable peristalsis vs. 5 in the group without; *p* = 0.5716, Mann–Whitney test). Additionally, 46 patients underwent 107 MAG3 scintigraphies (median: 2 per patient). Figure 1 illustrates the number of follow-up examinations in patients with and without initial detection of ureteral peristalsis.

Complete spontaneous resolution of the megaureter was observed in 66% (42/63) patients. Persistent dilatation of ≥7 mm was documented in 12.6% (8/63) of patients, six of whom were discharged from follow-up due to stable regression.

Surgical reimplantation was performed in 20.9% (13/63) resulting in complete resolution in 11 patients and significant improvement in 2.

The mean ureteral diameter was 11 mm (SD +/− 4.5 mm) with no difference concerning sex (*p* = 0.75, Mann–Whitney test). Univariate analysis showed no difference in the frequency of pyelonephritis or surgery associated with a ureteral diameter smaller or larger than 10 mm (*p* > 0.5, Fisher’s Exact test).

Peristalsis was detected in 33 patients (52.3%) during the initial examination. Peristalsis over follow-up was variably present with no clearly visible association with the fate of each single patient.

**Figure 2:**
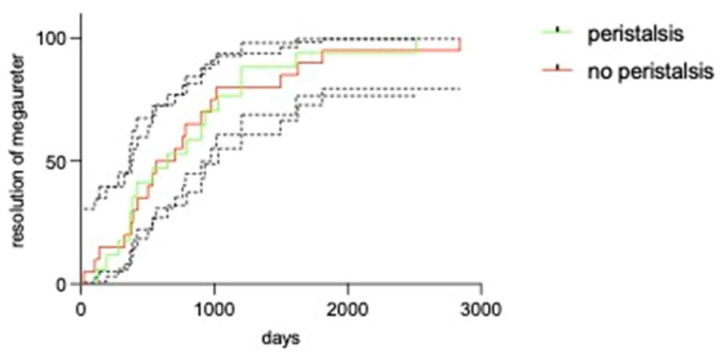
Resolution of megaureter over time in the group of non-operated patients. Shaded areas represent 95% confidence intervals for both groups.

Time to resolution of the megaureter was not different between those with or without peristalsis (*p* = 0.948; Mantel–Cox analysis), as shown in Figure 2.

To identify a potential trend, two extreme subgroups were compared: in patients who never had any peristalsis detected (n = 9), three underwent surgery whereas six showed spontaneous resolution of the megaureter. Conversely, in those where peristalsis was detected in each exam (n = 8), all megaureters were maturated.

During follow-up, a total of 49 episodes of pyelonephritis (12 under antibacterial prophylaxis) occurred in 42% (27/63) of patients. The diagnosis was prompted by pyelonephritis in six patients. Eleven episodes of breakthrough pyelonephritis during antibiotic prophylaxis occurred in 10 patients. Univariate analysis showed no influence peristalsis (*p* > 0.5, Fisher’s Exact test) concerning UTIs, UTIs during the first year of life or breakthrough UTIs during antibiotic prophylaxis.

In patients with initial peristalsis, spontaneous maturation of the megaureter was not more likely to occur (76.9% vs. 91.6%, *p* = 0.25, Fisher’s Exact Test). Surgery was not more likely in those without peristalsis (21.2% vs. 20%, *p* = 0.99, Fisher’s Exact Test). A logistic regression analysis with stepwise backward model selection, incorporating initial peristalsis and correcting for age, grade of ipsilateral hydronephrosis, ipsilateral ap diameter and sex, as well as looking at the diameter as a continuous variable, did not show any association with the predefined outcome. The initial model had 61 residual degrees of freedom; the final model had 50. Peristalsis showed a *p*-value of 0.54 in the initial model for UTI vs. no UTI, and 0.56 for surgery vs. resolution. Consequently, the effect was removed in the selection process.

## 4. Discussion

We demonstrated that visible peristalsis on an initial ultrasound is irrelevant to prognosis. Also, we showed that peristalsis as seen on ultrasound is a highly fugitive and inconsistent phenomenon during the follow-up of megaureters. Consequently, the presence of peristalsis should not be used as a parameter for clinical decision-making.

Diagnostic tools applied in patients with PM include sonography, mercaptoacetyltriglycine (MAG3) scans and voiding cystourethrography (VCUG). These often repeated examinations contribute to high morbidity for children and families. Especially during the first months of life, neither result has any consequence as antibiotic prophylaxis is considered the standard management during the first year of life due to the very high incidence of UTIs of up to 50% [15].

Reliable, predictive variables enabling the prediction of non-surgical management and timely intervention before loss of kidney function are scarcely available. A large ureteral diameter has been shown to correlate with the risk of undergoing definitive surgery [16,17,18]. The practical application of these findings is limited, however, as none of these studies included patients with homogeneous age at inclusion and no studies excluded all other pathologies related to megaureters. Clear cut-off diameters are not available.

Differential renal function (DRF) of the concerned renal moiety, and especially its loss over time, is an important argument in surgical indication; however, performing repeated MAG3 scintigraphies is invasive and associated with radiation burden.

The diagnostic burden imposed on children with congenital malformation of the urinary tract is significant: the 63 patients underwent a total of 107 scintigraphies, 63 VCUGs and 445 sonography exams—with the consequence that 13 underwent curative surgery. On the other hand, the associated morbidity, with 49 episodes of pyelonephritis concerning 27 patients, is no less significant. This consequence shows the dire need for parameters enabling prediction of the natural course to ensure either efficient prophylactic treatment or the omission of further diagnostic procedures. The presence of ureteral peristalsis can be easily observed on ultrasound and is therefore an apt candidate for further evaluation.

The observation of peristalsis on ultrasound per se—in a real-life setting—is a fugitive and not sufficiently reliable finding. The repeated observation of peristalsis on ultrasound over a long period of follow-up, being rare (n = 8 in this series), might be a better indicator of the further fate of these patients: none of the few patients with a constant presence of peristalsis on ultrasound underwent surgery (*p* > 0.001).

There is a consistent link between the presence of peristalsis and ureteral dilatation in the literature, starting in 1971, with a seminal, radiological publication by Pfister et al. (who later coined the eponymous megaureter classification) and continuing until, recently, a mathematical model was published that clearly demonstrates a reciprocal relationship between peristalsis and dilatation [6,8]. The classical theory suggested a distal aperistaltic segment was at the root of the presence of a more proximal megaureter. Also, in an animal model, differing responses to peristalsis-inducing transmitters were shown in megaureters compared to normal ureters—this is probably relevant to the resolution of PM and implies visible peristalsis is a sign of physiological function [19].

Therefore, the common notion that visible peristalsis is a “sign of hope” correlating with higher odds of resolution and potentially lower risk for UTIs seems not only logical but is also backed by theoretical scientific evidence. That this small but highly consistent series fails to show a clear impact, however, warrants cautious consequences after the observation of initial peristalsis on ultrasound: neither UTIs nor resolution differ in likelihood in patients with visible peristalsis.

UTIs are closely associated with megaureters. In this series, at least one and up to six episodes of pyelonephritis occurred in nearly half of all patients, even after the exclusion of all patients with VUR or associated pathologies, such as posterior urethral valves with an independent further risk for UTIs. This is highly congruent with one other, relatively large study (n = 212) where VUR was systematically excluded and 43% of all included patients suffered UTIs. However, this group included many patients diagnosed because of symptoms [15]. In Austria, neonatal circumcision is not routinely performed and does not represent a standard component of clinical practice; consequently, none of the patients in our cohort had been circumcised at the time of first presentation. Nevertheless, circumcision is offered to all patients who undergo surgical intervention for an underlying condition.

A potential limitation of our study is the sample size, which may limit the ability to detect small differences between patients with and without peristalsis at the time of the initial examination. Although we did not observe a relevant difference in the natural course between the groups, small effect sizes may have gone undetected. While a larger cohort might have allowed for the detection of statistically significant minor differences, the observed effect estimates suggest that any such differences would likely be small and of limited clinical relevance. Conversely, only through the exclusion of many potentially confounding factors can meaningful results be achieved—which, in this case, reduced the number of eligible patients from 308 to 63. Furthermore, as in a real-life clinical setting, we cannot exclude that the sonography was subject to a certain extent of subjectivity.

## 5. Conclusions

In this homogeneous cohort of patients with primary non-refluxing megaureter, ureteral peristalsis detected on ultrasound proved to be a highly transient and inconsistent finding, and was therefore not a sufficiently reliable parameter during follow-up. Consequently, the presence of peristalsis on initial ultrasound does not represent a useful predictive variable with regard to the frequency of pyelonephritis episodes or the likelihood of spontaneous resolution in infants under 1 year of age. Although peristalsis was systematically assessed, it showed no clinically relevant association with key outcomes, including spontaneous resolution or the incidence of urinary tract infections. As a result, this parameter does not provide additional value for risk stratification or clinical decision-making that would justify any modification to the current diagnostic or follow-up strategies.

## Figures and Tables

**Table 1:** Patient characteristics: (Group 1, based on no peristalsis during first US n = 30; Group 2, based on peristalsis during first US n = 33); MU—megaureter; SFU—Society of Fetal Urology.

	No Peristalsis *n* = 30	Peristalsis	
sex (f/m)	2f 28 m	13f 20 m	*p* = 0.001
median age at initial diagnosis	1.2 months (0.5–2.3)	1.6 months (0.7–3.4)	*p* = 0.47
median duration of follow-up	32 months (12–67)	36.6 months (17.3–56)	*p* = 0.85
side of MU (left/right/bilateral)	left 24 right 4 bilateral 2	left 13 right 12 bilateral 8	
no hydronephrosis		1	
SFU grade 1	2	2	
SFU grade 2	9	7	
SFU grade 3	13	16	
SFU grade 4	6	7	

## Data Availability

The raw data supporting the conclusions of this article will be made available by the authors on request. The data are not publicly available due to restrictions imposed by the ethics committee.
